# Pediatric Spinal Atypical Teratoid Rhabdoid Tumor: Recent Advances in Biology and Management Options

**DOI:** 10.3390/cancers18071171

**Published:** 2026-04-05

**Authors:** Ruby Siada, Kaushik Banerjee, Payal Malhotra, Mohannad Ibrahim, Daniel C. Moreira, John R. Prensner, Santhosh A. Upadhyaya

**Affiliations:** 1 Department of Pediatrics, University of Michigan, Ann Arbor, MI 48109, USA; rubsiada@med.umich.edu; 2 Department of Biological Chemistry, University of Michigan, Ann Arbor, MI 48109, USA; kaushb@med.umich.edu (K.B.); prensner@med.umich.edu (J.R.P.); 3 Department of Pediatrics, Division of Pediatric Hematology Oncology, University of Michigan, Ann Arbor, MI 48109, USA; 4 Department of Pediatric Hematology-Oncology & BMT, Rajiv Gandhi Cancer Institute & Research Centre, Delhi 110085, India; payalmalhotradr@gmail.com; 5 Department of Radiology, University of Michigan, Ann Arbor, MI 48109, USA; mibrahim@med.umich.edu; 6 Department of Oncology, Division of Neuro-Oncology, St. Jude Children’s Research Hospital, Memphis, TN 38105, USA; daniel.moreira@stjude.org

**Keywords:** atypical teratoid rhabdoid tumor, pediatric spinal cord tumor, AT/RT-MYC, intradural tumor

## Abstract

Atypical teratoid rhabdoid tumor (AT/RT) is a rare, highly aggressive embryonal central nervous system malignancy occurring predominately in infants and toddlers. Spinal AT/RT (spAT/RT) cases are even more limited, and as a result, little is known regarding prognostic factors and optimal treatment regimens. Molecularly, AT/RT is divided into three groups: AT/RT-SHH, AT/RT-TYR and AT/RT-MYC. spAT/RT is predominantly of the MYC subtype. Additionally, a third of patients with AT/RT have a germline Rhabdoid Tumor Predisposition Syndrome (RTPS) that increases the likelihood of developing additional rhabdoid tumors, including renal rhabdoid tumors. Due to the rarity of these tumors, there is a lack of consensus on treatment strategies to be employed. This review paper details the published literature on spAT/RT, with particular emphasis on the recent advances in understanding the biology of these aggressive tumors and currently available therapeutic options, and highlights the challenges associated with the management of this extremely rare condition.

## Simple Summary

This review article on primary spinal cord atypical teratoid/rhabdoid tumors (spAT/RT) in children presents a summary of the epidemiology, clinical presentation, management and recent advances in understanding the biology of AT/RT. AT/RT is a rare but highly malignant childhood tumor that occurs predominantly in the brain, with a very limited number of cases reported to occur primarily in the spinal cord. This has made evidence-based treatment recommendations of spAT/RT very challenging. Like its counterpart in the brain, spAT/RT in children requires trimodal therapy with surgery, chemotherapy, and radiation therapy to be cured, but outcomes remain dismal despite dose intensification. Recent advances in understanding the biology of AT/RT are opening the door for trialing biologically driven molecularly targeted therapies, including immunotherapies, that have the potential to redefine the management and outcomes of AT/RT.

## 1. Introduction

Atypical teratoid rhabdoid tumor (AT/RT) constitutes roughly 1–3% of pediatric brain tumors, and about 90% of cases occur before the age of three [1,2]. According to The Central Brain Tumor Registry (CBTRUS), the overall annual incidence rate of AT/RT is about 0.09 per 100,000, and incidence rates do not differ significantly by sex, race, or Hispanic ethnicity [3]. Within this rare disease group, primary spinal AT/RT (spAT/RT) comprise about 2–5% of tumors, and to date, there are about 50 cases reported in the literature [2,4,5,6,7,8,9,10,11,12,13,14,15,16,17,18,19,20,21,22,23,24,25,26].

The five-year overall survival (OS) for children with AT/RT ranges from 30% to 50%, with the best outcomes seen for patients with localized disease who underwent trimodal treatment [18]. Reports have noted that spAT/RT is associated with significantly worse outcomes. For the majority of pediatric patients with spAT/RT, OS is typically less than a year [4,5,19,21]. However, like its intracranial counterpart, long-term survival is feasible with multimodality therapies.

Due to the limited understanding of the clinical and biological behavior of these tumors, we undertook a comprehensive review of the literature on primary spAT/RT in children, adolescents and young adults (AYA), with an emphasis on presentation, evidence-based management, molecular underpinnings and future direction. The need for a focused review on spAT/RT was felt to be necessary due to the limited published data, especially from clinical trials that overwhelmingly enrolled children with intracranial AT/RT and the evolving biological underpinnings of this highly malignant childhood cancer, suggesting that spAT/RT may be a distinct biological entity compared to its intracranial counterparts.

## 2. Methods

We performed a search of PubMed using the following keyword combinations: “pediatric,” “spinal,” “teratoid,” and “rhabdoid.” This resulted in a total of 72 articles that were then reviewed for relevance. We included articles that reported on pediatric and AYA patients with primary spAT/RT lesions, including case reports, small case series and review articles. References cited from the initially reviewed articles were also included.

## 3. Clinical Presentation

AT/RTs mainly arise in supratentorial and infratentorial regions of the brain, but they can infrequently appear as primary spinal lesions. AT/RT is primarily a disease of infants and toddlers, with an overwhelming majority presenting under three years of age at the time of diagnosis; median age of presentation is around 32 months [14,24,25,27].

An intradural extramedullary location is the most common site of presentation when the spinal cord is the primary site of tumor. However, an intramedullary primary and, rarely, an extradural primary location may be noted in some patients [5,21]. The rates of intramedullary, intradural extramedullary, and extradural lesions vary among different studies. In a review of 58 spAT/RT pediatric patients, Li and colleagues reported that most children (53%) had intradural extramedullary lesions, followed by intramedullary and, less commonly, extradural lesions [19]. Extension through the intervertebral foramen has also been reported in some cases. Similarly, a case series of four patients and review of the literature done in 2019 further supported that most tumors are intradural but extramedullary [5]. Some authors report that lumbar and thoracic spinal lesions are the most common, followed by sacral and cervical lesions; however, others have reported that the cervical spinal cord is the most predominant tumor site [5]. Tumors often involve multiple overlapping spinal segments [5,19,21].

Children with intracranial AT/RT present with metastatic disease in approximately a third of cases [14,24,25,27]. However, spAT/RT has been reported to have higher rates of leptomeningeal seeding and metastasis, with some cohorts noting up to almost half of patients presenting with metastatic disease at initial diagnosis [5,19,21,28,29]. It should be noted, however, that these cohorts are small in size, and the true incidence of metastatic disease at initial diagnosis remains unclear. Due to the potential elevated risk for metastatic disease at presentation, it is important to obtain complete CNS imaging, which should include contrast-enhanced MRI of the brain and total spine, and CSF cytology by lumbar puncture 10–14 days post-surgical intervention, unless medically contraindicated, to evaluate tumor seeding.

Like other spinal cord tumors, the clinical presentation of patients with spAT/RT is dependent on the anatomic location of the tumor and is typically secondary to mass effect on surrounding neurovascular structures. Pain, either local at the site of the tumor in the form of neck or back pain and/or neuropathic pain in the affected dermatomes, is often a predominant finding at presentation. Extremity weakness manifesting as gait instability due to lower limb weakness and regression of gross motor milestones in toddlers are accompanying motor manifestations. In the more severe cases, monoparesis, paraparesis or quadriparesis may ensue depending upon the rapidity of the growth. Bowel and bladder dysfunction are noted in a substantial number of patients but not in all [5,16,19,21].

## 4. Imaging

**Figure 1:**
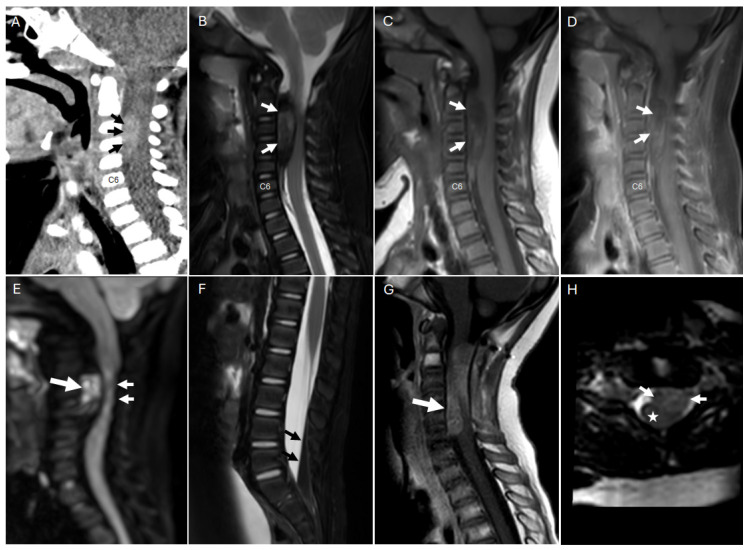
CT and MRI images of a cervical spAT/RT lesion in a 3-year-old patient: (**A**) Sagittal CT image of the cervical spine demonstrates slightly hyperdense mass in the anterior aspect of the cervical spinal canal (black arrows). (**B**) Sagittal T2W image demonstrating an intradural, extramedullary mass extending from C2 to C6 (white arrows), appearing heterogeneously isointense and hypointense relative to the spinal cord. (**C**) Sagittal T1W image demonstrating an intradural, extramedullary mass extending from C2 to C6 (white arrows), appearing heterogeneously isointense and hypointense relative to the spinal cord. (**D**) Sagittal post enhanced T1W image of the cervical lesion showing poor enhancement following contrast administration (white arrows). (**E**) Sagittal DWI image demonstrating intralesional restricted diffusion with high DWI signal indicating high cellularity (large arrow) with associated mass effect and posterior displacement of the spinal cord (small arrows). (**F**) Sagittal T2W image demonstrating layering hemorrhage in the distal spinal canal (black arrows). (**G**) Sagittal post-enhanced T1W image demonstrating enhancing lesion at the surgical bed surrounding the cord representing tumoral recurrence (white arrow). (**H**) Axial T2W image demonstrating isointense mass (arrows) displacing the cord posteriorly (star) at relapse.

The clinical and radiologic presentations of spAT/RT are frequently nonspecific, which contributes to diagnostic uncertainty and may delay definitive management. Imaging features often reflect the high cellularity and histologic heterogeneity of the tumor. Computed tomography (CT) imaging may show soft-tissue density lesion (Figure 1A), but diagnosis typically requires magnetic resonance imaging (MRI). On MRI, spAT/RTs typically appear as intradural masses with mixed signal intensity on T1-weighted (T1W) and T2-weighted (T2W) sequences, corresponding to solid, necrotic, cystic, and hemorrhagic components (Figure 1B–D) [30,31]. Diffusion restriction is frequently observed in the solid portions, reflecting the high cellularity of the lesion (Figure 1E) [32,33]. Intratumoral hemorrhage is relatively common, which appears as intralesional T1 hyperintense foci with susceptibility on GRE/SWI imaging. Given the tumor’s known propensity for leptomeningeal dissemination, imaging of the entire neuroaxis with and without contrast is recommended to assess for intracranial or spinal spread. In the patient referenced in the figure below, brain MRI performed at presentationn revealed no supra- or infratentorial lesions, aside from the cervical mass and layering subarachnoid hemorrhage within the spinal canal (Figure 1F), supporting the diagnosis of a primary, hemorrhagic spinal AT/RT.

The imaging features of spAT/RT overlap with other pediatric spinal tumors, contributing to diagnostic difficulty. Differential diagnoses based on imaging alone are broad, including ependymoma, other embryonal tumors, metastatic disease, astrocytoma, and other intradural neoplasms, as no imaging features are pathognomonic for spAT/RT [20]. Compared with ependymoma, which commonly arises intramedullary with associated hemorrhagic and cystic changes [30], spAT/RT more often appears heterogenous with pronounced diffusion restriction and necrotic components. High-grade astrocytoma, another intramedullary tumor, shows infiltrative growth patterns with indistinct margins, often with heterogeneous contrast enhancement and cystic areas [34]. Metastatic disease may mimic spAT/RT, particularly in the setting of drop metastases, but usually occurs in patients with known primary malignancy and often demonstrates multifocal nodular leptomeningeal enhancement. Intraspinal extension of Ewing sarcoma and neuroblastoma may occasionally manifest as large masses within the spinal canal; however, identification of associated paravertebral component often suggests an extraspinal origin of these lesions. While no imaging features are pathognomonic, the recognition of patterns such as intradural-extramedullary location, heterogeneous signal, diffusion restriction, and hemorrhagic foci may help raise suspicion for spAT/RT, but histopathologic and molecular confirmation remains essential for definitive diagnosis [30]. Early local recurrence and leptomeningeal dissemination are frequently reported, often occurring within months of initial diagnosis (Figure 1G,H). This aggressive clinical course highlights the need for close surveillance with serial MRI of the entire neuroaxis during and post-completion of therapy at a frequency of not less than every 2–3 months or in the case of concerning symptoms.

## 5. Biological Features of AT/RT and spAT/RT

The majority of AT/RT cases are defined by bi-allelic loss of function mutations in the *SMARCB1* gene on chromosome 22q11.2, and the remaining small minority of cases are born from loss of function mutations in the *SMARCA4* gene on chromosome 19p13.2 [35]. Both genes encode proteins that function as subunits of the SWItch/Sucrose non-fermentable (SWI/SNF) chromatin remodeling complex [36]. When these mutations are present in the germline, they result in Rhabdoid Tumor Predisposition Syndrome (RTPS). While the germline presence of *SMARCB1* alteration is termed RTPS-1, the presence of that of *SMARCA4* is termed RTPS-2 [7,37]. A majority of these germline alterations are de novo and lack a family history of similar predispositions. The incidence of RTPS has been found to be variable but most studies report an approximate incidence of 30% in all patients with AT/RT [24,25,38]. Additionally, rhabdoid tumors may occur synchronously or metachronously in children with underlying RTPS [7]. Hence, the possibility of these patients with extramedullary AT/RT being a subset of extra-CNS malignant rhabdoid tumor (MRT) has also been raised [21].

Tumor DNA methylation and transcriptome profiling have demonstrated that *SMARCB1*-mutant AT/RTs can be classified into three major molecular subtypes: AT/RT-MYC, AT/RT-SHH, and AT/RT-TYR [39,40]. The three subgroups show distinct clinico-pathologic correlation and also *SMARCB1*/chromosome 22 alteration patterns. The prognostic significance of the three different subtypes remains to be determined. While the AT/RT-TYR tumors demonstrate upregulation of proteins in melanosomal pathway, the AT/RT-SHH tumors have an overexpression of proteins in SHH and NOTCH pathways. The MYC subgroup is characterized by an overexpression of the *MYC* oncogene in addition to high expressions of *HOTAIR* and other HOX cluster genes [39,40,41].

Reports thus far show that the vast majority of spAT/RTs are of the AT/RT-MYC subgroup [19,21]. Because there is scant literature on the biological underpinnings of spAT/RT specifically, as well as lack of spAT/RT-specific disease models for mechanistic work, the scientific basis of this disease is largely extrapolated from studies in intracranial AT/RT. Importantly, accumulating evidence from DNA methylation datasets, together with increased expression of HOX and other mesenchymal developmental genes, indicates substantial biological similarity between AT/RT-MYC and extracranial malignant rhabdoid tumors (MRT/ECRT). These findings support the hypothesis that spAT/RT may have unique biological features compared to other forms of intracranial AT/RTs [42].

At the mechanistic level, spAT/RT is characterized by the loss of *SMARCB1* (*INI1*), which promotes malignant transformation through epigenetic dysregulation and an increased dependence on EZH2/EZH1-mediated trimethylation of histone H3 at lysine 27 (H3K27). *SMARCA4* mutations have not been reported in pediatric spinal AT/RT, but a case of an adult patient with *SMARCA4*-mutant spAT/RT has been published, though this is likely to be extremely rare [43]. Recently, knowledge of AT/RT-MYC subtype biology has been enhanced due to the plethora in vitro AT/RT models derived from AT/RT-MYC patients, including the BT12, BT16, CHLA-06-ATRT, and CHLA266 cell [35]. Multiple in vitro drug screens have investigated a connection between the molecular biology of AT/RT-MYC tumors and targeted therapies. Here, multiple studies have highlighted a potential role for multi-tyrosine kinase inhibitors in AT/RT-MYC [44,45,46], with a single study also identifying a CDK4/6 and eukaryotic translation initiation factor, briciclib [47]. Uniquely, SWI/SNF disruption in AT/RT-MYC tumors appears to have substantial impacts on the tumor microenvironment (TME), which may be the most distinguishing feature of AT/RT-MYC compared to AT/RT-SHH tumors. AT/RT-MYC tumors are considered immunologically “hot,” with high expression of immune gene signatures [35]. Indeed, the AT/RT-MYC TME is highly infiltrated by lymphocytes, many of which are cytotoxic T-cells or exhausted effector memory CD8+ T-cells. This is accompanied by the high expression of immune checkpoint genes *PD1* and *PD-L1*, as well as terminal differentiation and IFNα-response gene signatures [42,48]. Notably, this area of research is now influencing the next generation of clinical trials for AT/RT. For example, several studies suggest that *SMARCB1*-deficient tumors may benefit from immune checkpoint inhibition [49,50]. The TAZNI clinical trial (tazemetostat plus nivolumab and ipilimumab, NCT05407441) test this directly in the pediatric AT/RT population, although this will be contingent on the availability of tazemetostat in the future [51].

Identifying therapeutic targets related to the AT/RT-MYC TME has been a priority area for research. Importantly, the AT/RT-MYC T-cell populations appear to be clonally derived, suggesting that they have expanded in response to an antigen stimulus [42]. While the antigen basis for T-cell expansion remains an area of investigation, intriguing findings point to a possible role for endogenous retroviral proteins, which can activate innate immune response genes [42]. Indeed, *SMARCB1* deletion is a causal factor for human endogenous retrovirus (HERV) reactivation, and MYC specifically binds to the HERV-K long terminal repeat (LTR) in AT/RT [52]. However, antigens derived from canonical proteins may also play a role as well. For example, the gene Claudin 6 (*CLDN6*), which is normally suppressed outside of fetal tissues, is reactivated in AT/RT-MYC and serves as a T-cell target, including therapeutic chimeric antigen receptor T-cells (CAR-T) designed to recognize the CLDN6 antigen [53]. The biological basis of AT/RT, including spAT/RT, has therefore expanded rapidly, providing exciting opportunites for translation into clinical trials.

## 6. Diagnosis

AT/RT is a heterogenous tumor of embryonal origin with a poly-immunophenotype that characteristically has a population of rhabdoid cells intermixed with primitive neuroectodermal, mesenchymal and epithelial cells giving it the “teratoid” “rhabdoid” nomenclature [41,54]. While rhabdoid cells are the hallmark finding, it is to be noted they may be the exclusive or predominant finding in only a small proportion of cases and may even be absent in some cases, making the diagnosis a challenging one and one that requires a high index of suspicion [55]. The variable components of AT/RT result in a broad range of immunoreactivity including positivity for EMA, SMA, Vimentin, GFAP, NFP and synaptophysin, while germ cell markers and skeletal muscle markers may be absent. However, the finding of bi-allelic loss of *SMARCB1* in an overwhelming majority of AT/RT cases with resultant nuclear loss of SMARCB1 or INI1 expression has resulted in a highly sensitive marker for AT/RT diagnosis in the presence of other typical or, rarely, atypical histological findings [56]. In the remaining cases, the loss of nuclear expression of SMARCA4 or BRG1 protein is diagnostic. The retained expression of INI1 or BRG1 on the vascular endothelial cells in the tumor serves as an internal control [57]. In the rare cases of diagnostic ambiguity despite extensive testing, DNA methylation testing is indicated for accurate diagnosis [39]. It is pertinent to note that the loss of INI1 is not exclusive to AT/RT and may be found in other CNS tumors like cribriform neuroepithelial tumors, chordoma and desmoplastic myxoid tumor of pineal origin, SMARCB1-deficient [58,59,60]. Within the spinal cord and vertebral coloumn area, INI1 loss may be seen in poorly differentiated chordoma and schwannomatosis associated schwannomas [60,61,62]. Hence, due caution must be exercised during the diagnostic steps, taking into consideration the age of the patient, the tumor location and the histopathologic findings while diagnosing AT/RT.

All patients diagnosed with AT/RT are recommended to undergo germline testing to rule out RTPS with gene analysis of *SMARCB1* or *SMARCA4* depending upon the corresponding tumor finding. However, it should be noted that, as stated above, spAT/RT are often of the MYC subtype and two large studies suggest a very low incidence of germline predisposition in this subtype of AT/RT compared to AT/RT-SHH and the AT/RT TYR subtypes [24,25]. This finding needs to be confirmed in the future with larger data sets.

## 7. Treatment

### 7.1. At Initial Diagnosis

AT/RT treatment is often trimodal, consisting of surgery, chemotherapy, and radiation [24,25,27,63]. Due to the rarity of spAT/RT, there is no consensus on a standard-of-care treatment for these patients. It has been shown in some studies that gross total resection (GTR) can improve progression-free survival (PFS) and overall prognosis for AT/RT in an intracranial location [64,65]. However, other studies and clinical trials have shown that the outcome was not significantly affected by the extent of the resection [27,66]. Additionally, based on the location of the tumor, GTR may not be a therapeutic possibility, and instead, maximal safe resection is pursued. The timing, dose, and field of RT (craniospinal [CSI] vs. focal) remains controversial and varies per protocol [67]. The side effects of CSI in patients this young can be significant and must be considered when approaching treatment strategies. However, earlier clinical trials that excluded radiation from treatment protocols had poor outcomes [68,69,70]. Hence, trimodal therapy incorporating surgery, chemotherapy, and radiation therapy has become the consensus management approach for intracranial AT/RT at most centers across the world. However, the optimal chemotherapy regimen to be used and the field of radiation to be employed in spAT/RT remains unclear.

The only relatively “large” study to date to report exclusively on the outcomes of children with spAT/RT was the multi-institutional retrospective study conducted under the EU-RHAB study group that described clinical and molecular characteristics, treatment, and outcome of children with spAT/RT collected over a 14-year period. In this report by Benesch and colleagues, the two-year progression-free survival (PFS) was only 26% and overall survival was 23% at a median study period of eight years for the 13 patients using multi-modality therapy. The majority of the patients were of AT/RT-MYC subtype with a median age of presentation of 32 months and almost half of them (six of the 13) presenting with metastatic disease (Table 1). While all patients received post-operative chemotherapy, only six patients received radiation therapy, CSI in three and focal in the other three patients [21]. Apart from this study, data on outcome is limited to only case reports or relatively small case series.

The major AT/RT trials including the St. Jude protocols (SJMB03 and SJYC07), EU-RHAB AT/RT, children’s oncology group study ACNS0333, and DFCI-AT/RT have very limited data on primary spAT/RT cases (Table 1) [14,24,27,66]. In fact, of these trials, only SJMB03 and ACNS0333 provide reference to patients with spAT/RT. SJMB03 enrolled children over three years of age and stratified patients into average risk vs. high risk based on degree/presence of metastasis and/or residual tumor. All patients in this trial underwent CSI (average-risk patients received 23.4 Gy of CSI followed by focal radiation to the primary tumor bed with a total dose of 55.8 Gy; high-risk patients received 36–39.6 Gy of CSI followed by focal radiation to the primary tumor bed with a total dose of 55.8–59.4 Gy). After CSI, patients were given a six-week rest followed by four cycles of multi-agent high dose chemotherapy (cisplatin, cyclophosphamide, and vincristine) followed by autologous stem cell transplantation. The primary endpoints for this study were five-year OS and PFS; however, the two spAT/RT patients within this cohort did not live beyond five years [24]. ACNS0333 combined two cycles of intensive induction multi-agent chemotherapy (methotrexate, vincristine, etoposide, cyclophosphamide, and cisplatin) with three cycles of consolidation (thiotepa and carboplatin) followed by autologous stem cell rescue. In addition to chemotherapy, second-look surgery was encouraged prior to consolidation if there was evidence of resectable disease. The order of radiation and consolidation was based on tumor location, extent of disease, and age. The protocol allowed for either photon or proton therapy, and the total dose of radiation to the primary site was dependent on the patient’s age (50.4 Gy for patients less than three years old and 54 Gy for patients older than three years). Within this study, only one case of spAT/RT was included, and the survival data was combined with patients who had both infratentorial and supratentorial lesions [27].

### 7.2. Treatment of Recurrent or Relapsed Disease

As detailed earlier, there is a lack of consensus on the optimal management of AT/RT at initial diagnosis. Needless to say, there is even less published data and consensus on successful management of relapsed or progressive (PD) AT/RT that is refractory to frontline therapies. In the case of spAT/RT, this is compounded further by rarity of the condition. Consequently, the literature review on salvage therapies will primarily highlight AT/RT treatment in general, with reference to spAT/RT subjects if any were included. It is important to acknowledge that this does limit the therapeutic conclusions for patients with spAT/RT, and extrapolating from AT/RT is an imperfect, albeit necessary action given the state of the currently published literature.

In a single institution study from St. Jude Children’s Research Hospital of recurrent/refractory AT/RT (PD), only five of 64 children were alive at a median follow up of 10.9 years from PD with a five-year OSpostPD of 7.3% for the entire cohort and two-year OSpostPD in those receiving any salvage therapy (n = 39) of 33%. Due to the limited number of long-term survivors and heterogeneous treatment employed, no recommendations were made on specific salvage therapy regimens. However, it is notable that salvage RT, either focal or CSI, and ICE chemotherapy were utilized in some of the survivors [73,74]. There were three subjects with spAT/RT in this study and all three (AT/RT-MYC subtype) were deceased within six months from the date of relapse (Carey S et al.; personal communication). Shiba and colleagues reported on survival using bevacizumab, irinotecan and temozolomide with and without stereotactic radiotherapy in three children with relapsed/refractory AT/RT and one patient with CNS embryonal tumors with rhabdoid features. Even though the objective response rate-complete and partial response (CR + PR) was 75.0% in the four patients, all three patients with AT/RT had passed away from the disease at the time of reporting after having received 2–6 cycles of treatment, highlighting the significant challenges in treating children with relapsed AT/RT [75]. Peyrl and colleagues reported the outcomes of children with recurrent embryonal tumors who were treated with multi-agent antiangiogenic therapy and intrathecal chemotherapy (modified MEMMAT), in which three children with AT/RT, including one with multiple recurrent tumors, were alive after 10 to 42 months. Neither of the above two studies have reported the presence of patients with spAT/RT [76]. It is hence challenging to draw conclusions, if any, on the efficacy of these salvage therapies, specifically in children with recurrent/relapsed spAT/RT. These systemic therapeutic options, however, remain available for select patients with relapsed spAT/RT.

As discussed above, *SMARCB1* or *SMARCA4* alterations are fundamental to AT/RT biology and oncogenesis, leading to loss of the SWI/SNF mediated inhibition of EZH2 and consequently decreased repressive marker, H3K27me3. Tazemetostat is a selective EZH2 inhibitor and is FDA approved in the United States for adult and pediatric patients with epithelioid sarcoma. In the NCI-COG pediatric MATCH APEC1621C phase 2 study of Tazemetostat for patients aged 1–21 years with refractory AT/RT, alongside other tumors harboring alterations in *EZH2* or members of the SWI/SNF complex, among eight treated patients with AT/RT, only one had prolonged disease stabilization beyond six months [77]. Additionally, the expansion phase of the phase I multicenter pediatric study of Tazemetostat had five of 21 ATRT patients with an objective response, with the median duration of response being 6.5 months. Hence, the benefit, if any, from Tazemetostat is largely transient in children with AT/RT. A multi-institutional study of aurora kinase inhibitor, Alisertib, led by St. Jude Children’s Research Hospital had a total of 30 enrolled patients with recurrent AT/RT receiving Alisertib once daily on Days 1–7 of a 21-day cycle till further PD occurred [78]. Although the study did not meet predetermined efficacy end point, single-agent Alisertib was well tolerated by children with recurrent AT/RT, and stable disease or partial response (SD + PR) was observed in approximately a third of the patients, thus demonstrating transient benefit or bridging some of the treated children to other therapies [78]. Neither trial has reported separately on spAT/RT participants nor made a mention of the anatomic location of the tumor in study participants. Another targeted therapy with limited data and success is the CDK4/6 inhibitor Ribociclib that was studied in a multi-center phase I study, with two of 13 patients with AT/RT showing stabilized disease and remaining on treatment for 20 and 24 months [79].

In addition to molecularly targeted therapies, another avenue for treating cancers is engineering patients’ immune system to attack cancer cells. Pre-clinical work by Theruvath and colleagues in cerebral AT/RT xenografts using B7-H3.BB.z-chimeric antigen receptor (CAR) T-cells administered intracerebroventricularly or intratumorally demonstrated potent antitumor effects making B7-H3 as a compelling target for the treatment of AT/RT and other tumors that express B7-H3 [80]. While immunotherapy strategies using CART cell therapy hold promise in recurrent brain tumors including AT/RT, they are still considered experimental, with several ongoing trials whose results are awaited (NCT04185038, NCT03638167, NCT04897321, NCT04510051).

Thus, children with recurrent/relapsed AT/RT and those with spAT/RT in particular have extremely limited therapeutic options and a vast majority succumb to their disease. Nevertheless, in select cases with individualized therapy plans, long-term survival is possible, albeit in very low numbers.

## 8. Management Challenges in Low- and Middle-Income Countries

The management of spAT/RT in low- and middle-income countries (LMICs) remains particularly challenging due to the tumor’s aggressive biology and the requirement for timely, intensive multimodal therapy. Similar to the data from rest of the world, reports of spAT/RT from LMICs is very limited and mostly presented combined with intracranial AT/RT outcomes [81,82]. In LMIC settings, however, multiple systemic barriers continue to compromise effective management of central nervous system tumors. Delayed referral pathways, limited availability of advanced neuroimaging, and restricted access to specialized pediatric oncology and neurosurgical centers contribute to diagnostic delays and advanced disease at presentation. Consequently, many patients present with advanced disease, substantially narrowing curative treatment options [82,83,84].

Accurate diagnosis of spAT/RT requires immunohistochemical confirmation of INI1 loss and occasionally molecular characterization. Many LMIC centers lack access to these diagnostic modalities. Consequently, tumors may be misclassified as other embryonal malignancies, resulting in non-AT/RT-specific treatment approaches and inferior outcomes [85].

Children with AT/RT in LMIC are treated with a mix of adapted and non-standard protocols, including modified DFCI IRS- III protocol, ATRT-2006, MUV-ATRT, EU-RHAB, and HIT-SKK-based approaches [86,87]. Data from LMICs consistently demonstrate poor outcomes for children with AT/RT despite multimodal therapy. In a study from Egypt, the authors reported median overall survival of approximately 10.3 months, with high rates of treatment-related toxicity and mortality, highlighting the limitations of delivering intensive chemotherapy in resource-constrained settings [81]. Ismail and colleagues recently reported on clinical and molecualr characetristics of 100 AT/RT patients, including four patients with primary spAT/RT, from a single center in Egypt that were molecularly annotated (n = 64) and treated uniformly on modified DFCI IRS-III protocol. Treatment-related mortality was high at 28% with a five-year OS of only 13% [82].

Similarly, experience from Mexico and other South American centers, albeit limited to small case series, report poor survival and emphasize delayed diagnosis and restricted access to comprehensive care as major determinants of outcome [88,89,90]. Data from the Indian subcontinent reflect comparable challenges, with reported median OS of 10 months despite multimodal therapy [91]. In contrast, data from Middle Eastern LMIC-adjacent settings show a median overall survival approaching 17 months with trimodality therapy underscoring the impact of access to complete treatment. Collectively, these studies suggest that while AT/RT outcomes in LMICs remain inferior to those reported in high-income countries, improved survival is achievable in select centers underscoring the impact of evolving infrastructure, multidisciplinary care, and better access to standardized multimodal therapy [92].

Surgical management in LMIC settings is frequently constrained by infrastructure and workforce limitations, shortages of experienced pediatric neurosurgeons, lack of intraoperative neurophysiological monitoring, and limited postoperative critical care support. Moreover, chemotherapy delivery and supportive care presents additional challenges. Standard AT/RT treatment protocols involve intensive multi-agent chemotherapy including tandem transplants and are associated with significant hematologic and infectious toxicity. Radiotherapy is a critical component of disease control in AT/RT; however, its delivery in LMICs is frequently compromised by systemic and patient-related factors including delayed initiation, unplanned treatment interruptions, incomplete radiotherapy courses, limited availability of functional radiotherapy units, and the need for patients to travel long distances for daily treatment [82]. This results in inferior local control of the disease and compromised survival. Due to the cumulative impact of these constraints and the historically poor prognosis associated with AT/RT, treatment is frequently perceived as non-feasible [93]. As a result, many patients are deemed palliative from the time of diagnosis or early on in treatment, either due to physician assessment of limited therapeutic benefit or family decisions influenced by socioeconomic burden, anticipated toxicity, and restricted access to comprehensive care [13].

Despite these limitations use of tri-modality therapy for the treatment of spAT/RT should be considered in LMIC settings. Among these, DFCI IRS-III, the St. Jude SJYC07 or the EURHAB treatment protocol for non-metastatic disease is implementable due to lower toxicity rates compared to HDCT protocols and, in the authors opinion, should be attempted with appropriate supportive care. Outcomes for those with metastatic AT/RT are very poor and it would be reasonable not to offer aggressive therapies, but instead palliative and supportive care be prioritized for these patients

## 9. Future Directions and Conclusions

Primary spinal AT/RTs are extremely rare but highly malignant tumors, that predominate in childhood and, due to their location, rarity and young age at presentation are often difficult to suspect and diagnose. Definitive diagnosis can be accomplished by histopathological characterization and immunohistochemistry staining for INI1 (in majority) or BRG1 (small minority) of tumor sample obtained by biopsy or maximal safe resection. However, limited reports of treatment modalities employed makes it challenging to identify a universal standard of care approach. Despite this, the availaible literature suggests that succesful treatment and long-term survival is achievable in select children by timely diagnosis, neurosurgical intervention and adjuvant chemo-radiotherapy similar to intracranial AT/RT. Metastatic disease at presentation is a poor prognostic factor in AT/RT with studies suggesting that childen with spAT/RT may have a higher incidence of metastases at diagnosis. Whether this clinical risk factor is primarily responsible for reported poor outcomes with spAT/RT or the biology of the AT/RT-MYC group that predominates in spAT/RT accounts for the dismal prognosis remains to be elucidated. Notably, AT/RT-MYC tumors are thought to have an immunologically “hot” tumor microenvironment, which may have implications for the application of immunotherapies in the spAT/RT patient population. Given the complexity of diagnosis and treatment, children in low–middle income countries are at particular disadvantage with reportedly higher mortality in these countries prompting the need for increased awareness on its timely diagnosis and management. Recent advances in understanding the biological basis of spAT/RT has opened up avenues for developing well designed clinical trials using molecularly targeted and immunotherpies aimed at AT/RT-MYC subtype. Future steps should include larger international collaborations through multi-instituional or consortium studies, development of clinical trials that combine molecularly targeted therapies with conventional trimodal therapies and optimizing resources in LMIC settings to improve survival in this rare but highly challenging childhood tumor.

## Figures and Tables

**Table 1:** Details of different protocols used in the management of AT/RT.

Protocol/Study Names	Number of Total Patients	Number of spAT/RT Patients	Treatment Regimen (Surgery, Chemotherapy, and Radiation)	Survival Outcomes
**Head Start III** [68]	19 M0 = 11 M+ = 8	0	Surgery: Initial maximal safe surgical resection with second look surgery following induction cycles for those with residual disease Systemic chemotherapy: Induction cycle 1, 3 & 5: CDDP/VCR/ETO/CPM/HDMTX Induction cycle 2 & 4: TMZ/ETO/VCR/CPM One cycle of high dose chemotherapy (CBCDA/TSPA/ETO) followed by autologous stem cell rescue RT: Delayed, reduced or avoided and only administered to children 6–10 years old or <6 if residual disease existed following chemotherapy (5 patients in total)	3 year EFS: 21% ± 9% 3 year OS: 26% ± 10%
**CCG9921** [71]	28	0	Surgery: Safe maximal at diagnosis, second surgery encouraged if residual tumor persisted after induction chemotherapy Systemic Chemotherapy Regimen A: VCR, CDDP, CPM, ETO × 5 cycles Regimen B: VCR, CBCDA, I, ETO × 5 cycles Maintenance: VCR, ETO, CBCDA, ETO × 8 cycles RT Persistent disease after induction/metastases at diagnosis: RT at 3 yr or following maintenance chemotherapy Progression or recurrence at any age (11 patients in total; 2 adjuvant and 9 salvage)	5 year EFS: 14%± 8% 5 year OS: 29%± 9%
**DFCI** **IRS-III** [14]	20	0	Surgery: Maximal safe tumor resection Systemic Chemotherapy: 51 weeks of VCR/ACT-D/CPM/CDDP/A/TMZ Weeks 1–6: pre-irradiation therapy Weeks 7–12: chemoradiation induction therapy Weeks 13–18: postradiation induction therapy Weeks 19–44: maintenance Weeks 25–51: continuation therapy +/− A Intrathecal chemotherapy: MTX, ARA-C, hydrocortisone Patients with initially positive CSF received weekly IT chemotherapy until two consecutive negative CSF; then as scheduled for patients with M0 disease. IT administration alternated between the intra-lumbar and intraventricular routes (when possible) RT: Following 6 weeks of induction chemotherapy, patients received concurrent radiation with chemotherapy. Patients with M0 disease received focal radiation; patients older than 3 years and M+ disease received CSI (15 patients in total)	2 year PFS: 53% ± 13% 2 year OS: 70% ± 10%
**EU-RHAB** [66]	35	0	Surgery: Maximal safe tumor resection followed by second look surgery (after at least 2 cycles of chemotherapy) for those with residual disease Systemic Chemotherapy: Post-operative chemotherapy: total of 9 courses (alternating VCR/CPM/A (5 courses) and I/CBCDA/ETO (4 courses) every 3 weeks Maintenance: 8 cycles of TRO/IDA and TRO/ETO every 3 weeks Optional HDCT: 19 patients received HDCT with autologous stem cell transplantation as either upfront therapy (N = 12) or at progression (N = 7) at the provider’s discretion Intrathecal chemotherapy: MTX or MTX/ARA-C/hydrocortisone administered via Ommaya or Rickham reservoir concurrently with systemic chemotherapy until start of radiation RT: Focal RT recommended for >18 months of age (23 patients); preferably after the 4th course of chemotherapy. CSI recommended for patients with M1–M4 disease ≥ 3 years (2 patients in total)	5 year EFS: 30.5 ± 4.2% 5 year OS: 34.7 ± 4.5% The results from HDCT were published separately, but due to a small, heterogeneous sample, no conclusion regarding HDCT efficacy could be made [72]
**ACNS033** [27]	65	1	Surgery: Maximal safe tumor resection followed by second look surgery after induction for those with residual disease Systemic Chemotherapy: Induction: Two 21-day cycles of VCR/MTX/ETO/CPM/CDDP Consolidation: 3 cycles of CBCDA/TSPA Followed by autologous stem cell rescue RT: Focal RT administered between induction and consolidation to patients at least 6 or 12 months old with tumors localized to the infratentorial or supratentorial brain, respectively. RT was given after consolidation for younger patients or those with metastatic disease. CSI was recommended at the discretion of the treating institution if patient was ≥3 years (42 patients in total)	4 year EFS: 37% 4 year OS: 43%
**SJYC07** [24]	52	0	Surgery: Maximal safe tumor resection followed by second look surgery after induction for those with residual disease Systemic Chemotherapy: IR Induction: 4 cycles of HDMTX/VCR/CPM/CDDP Maintenance: PO CPM/TOPO alternating with PO ETO HR Induction: 4 cycles HDMTX/VCR/C/CDDP/VBL Consolidation: 2 cycles CPM/TOPO Maintenance: PO CPM/TOPO alternating with PO ETO RT: IR: Focal RT HR:Optional CIS if ≥3 years old at the end of induction	IR: 5 year PFS: 31.4% ± 9.2% 5 year OS: 43.9% ± 9.5% HR: 5 year PFS and OS: 0%
**SJMB03** [24]	22	2	Surgery: Maximal safe tumor resection RT: All patients received risk-adapted CSI immediately following surgery Systemic Chemotherapy: Post-RT 4 cycles of CDDP/VCR/CPM followed by autologous stem cell rescue	AR: 5 year PFS: 72.7% ± 12.7% 5 year OS: 81.8% ± 11.0% HR: 5 year PFS and OS: 18.2% ± 9.5%
**Benesch et al. European Cohort** [21]	13	13	Surgery: Maximal safe resection/Biopsy Systemic chemotherapy: 4 patients treated via EU-RHAB 2 patients treated via Rhabdoid 2007 7 patients received different chemotherapeutic combinations on an individual basis including: CBCDA/ETO, CAV, I/CBCDA/ETO, VCR/CDDP/A/ETO, CPM/VCR/CDDP/A/ETO, TMZ, VBL Intrathecal chemotherapy: 3 patients (MTX, *n* = 2; MTX/cytarabine/prednisolone, *n* = 1) RT: 3 patients underwent local RT 3 patients underwent CSI irradiation	2 year PFS: 26.4% ± 12.9% 2 year OS: 23.1% ± 11.7%

Abbreviations: CDDP, cisplatin; CBCDA, carboplatin; VCR, vincristine; ETO, etoposide; CPM, cyclophosphamide; HDMTX, high-dose methotrexate; TMZ, temozolomide; TSPA, thiotepa; ACT-D, dactinomycin; A, doxorubicin; MTX, methotrexate; ARA-C, cytarabine; I, ifosfamide; TRO, trofosfamide; IDA, idarubicin; TOPO, topotecan; VBL, vinblastine; vincristine/doxorubicin/cyclophosphamide (CAV); High-dose chemotherapy, HDCT; RT, radiation therapy; CSI, craniospinal irradiation; EFS, event-free survival; PFS, progression-free survival; OS, overall survival; IR, intermediate risk; HR, high risk; AR, average risk.

## Data Availability

The original contributions presented in this study, if any, are included in the article. Further inquiries can be directed to the corresponding author.
